# Proteomics analysis of Schwann cell-derived exosomes: a novel therapeutic strategy for central nervous system injury

**DOI:** 10.1007/s11010-019-03511-0

**Published:** 2019-03-04

**Authors:** Zhijian Wei, Baoyou Fan, Han Ding, Yang Liu, Haoshuai Tang, Dayu Pan, Jiaxiao Shi, Pengyuan Zheng, Hongyu Shi, Heng Wu, Ang Li, Shiqing Feng

**Affiliations:** 1 Department of Orthopedics, Tianjin Medical University General Hospital, Tianjin, 300052 China; 2 Tianjin Key Laboratory of Lung Cancer Metastasis and Tumor Microenvironment, Tianjin Lung Cancer Institute, Tianjin Medical University General Hospital, Tianjin, 300052 China; 3 Department of Orthopedics, Henan Provincial People’s Hospital, Zhengzhou, 450000 China

**Keywords:** Schwann cell, Exosomes, Central nervous system, Proteomics

## Abstract

Exosomes are nanometer-sized vesicles involved in intercellular communication, and they are released by various cell types. To learn about exosomes produced by Schwann cells (SCs) and to explore their potential function in repairing the central nervous system (CNS), we isolated exosomes from supernatants of SCs by ultracentrifugation, characterized them by electron microscopy and immunoblotting and determined their protein profile using proteomic analysis. The results demonstrated that Schwann cell-derived exosomes (SCDEs) were, on average, 106.5 nm in diameter, round, and had cup-like concavity and expressed exosome markers CD9 and Alix but not tumor susceptibility gene (TSG) 101. We identified a total of 433 proteins, among which 398 proteins overlapped with the ExoCarta database. According to their specific functions, we identified 12 proteins that are closely related to CNS repair and classified them by different potential mechanisms, such as axon regeneration and inflammation inhibition. Gene Oncology analysis indicated that SCDEs are mainly involved in signal transduction and cell communication. Biological pathway analysis showed that pathways are mostly involved in exosome biogenesis, formation, uptake and axon regeneration. Among the pathways, the neurotrophin, PI3K-Akt and cAMP signaling pathways played important roles in CNS repair. Our study isolated SCDEs, unveiled their contents, presented potential neurorestorative proteins and pathways and provided a rich proteomics data resource that will be valuable for future studies of the functions of individual proteins in neurodegenerative diseases.

**Electronic supplementary material:**

The online version of this article (10.1007/s11010-019-03511-0) contains supplementary material, which is available to authorized users.

## Introduction

Central nervous system (CNS) injury, such as spinal cord injury and brain injury, causes irreversible loss of motor and sensory function [1, 2]. Due to the development of modern society, the occurrence rate of CNS damage increases year by year. The predicament of CNS regeneration is always attributed to the poor regenerative plasticity of mature neurons as well as the detrimental microenvironment caused by the lesion [3]. The peripheral nervous system (PNS) exhibits adequate regeneration after injury, which is different from the CNS. During this process, glial cells in situ play a more important role than functional neurons. In the CNS, multiple growth inhibitory factors derived from oligodendrocytes, such as Nogo, myelin-associated glycoprotein (MAG), and oligodendrocyte myelin glycoprotein (OMgp), induce the collapse of the growth cone [4]. Conversely, in the PNS, Schwann cells promote nerve regeneration through secreting growth factors, clearing myelin and axonal debris, activating macrophages and forming new medullary sheath [5].

Taking the advantages of Schwann cells into consideration, many studies have attempted to transplant these cells into injured CNS. This strategy obtained many encouraging results [6]. The advantages of Schwann cell transplantation include the practicability of autologous transplantation [7, 8] and synergistic effects combined with other strategies [9, 10]. However, some evidence still causes researchers to believe that Schwann cells transplanted into the CNS cannot perform as ideally as they can in the PNS. A major challenge for Schwann cell CNS transplantation is the low graft cell survival due to p75NTR-induced apoptosis [11, 12] and limited migration ability across the astrocyte boundary [13, 14]. Therefore, whether a previous favorable improvement is the mutual factor between active and adverse effects after SC transplantation in the CNS remains to be determined. We hypothesized that there should be a new strategy to optimize the ability of SCs to minimize undesirable characteristics.

Exosomes are small vesicles with a diameter of approximately 50–100 nm that are produced in the endosomal compartment and filled with functional proteins, microRNAs (miRNAs) and mRNAs [15]. They are involved in intercellular communication and are secreted continuously under many physiological and pathological conditions [16]. Their subparticle nature in the host cells makes them serve as microenvironment modulators through paracrine mechanisms to ease stimuli [17]. A recent study demonstrated that exosomes derived from Schwann cells support axonal maintenance and regeneration after PNS damage [18]. Considering the positive performance of SCs in CNS repair, we wondered whether the use of their exosomes is the optimal strategy to replace cell transplantation. Because of the complexity CNS composition, understanding exosome content is necessary to explore potential target cells. Until now, only individual proteins have been identified by various studies [19], which leads to the requirement of comprehensive information about the proteins in Schwann cell-derived exosomes (SCDEs).

In the current study, we purified exosomes from SC-conditioned medium and explored their morphological features and biomarkers. Furthermore, 433 proteins were identified from SCDEs using LC-MS/MS. Systematic proteomics characteristics were obtained through bioinformatics analysis. This study provides new evidence that SCDEs may act as a novel therapeutic strategy for CNS injury.

## Methods

### Isolation of exosome vesicles by ultracentrifugation

**Fig. 1:**
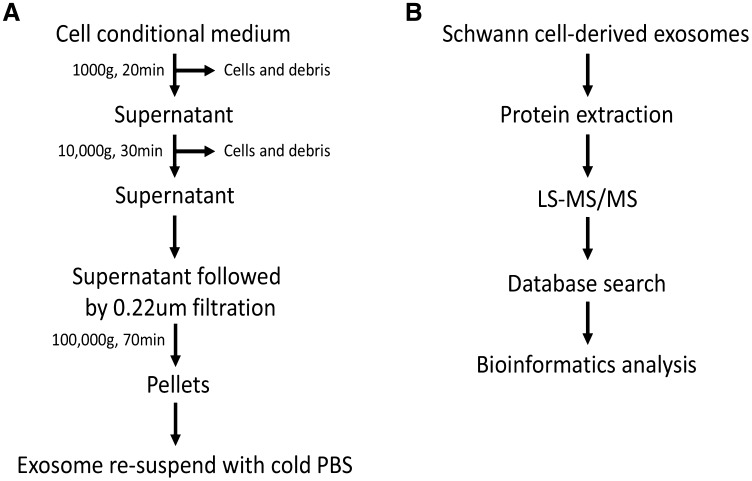
Isolation procedure of Schwann cell-derived exosomes and the workflow of proteomic analysis. **a** The isolation procedure of Schwann cell-derived exosomes (SCDEs) and **b** workflow for the processing of SCDEs for a comprehensive proteomic analysis

Primary SCs derived from the sciatic nerve of adult Wistar rats (female, 230 g ± 10 g, provided by the Academy of Military Medical Sciences, Tianjin, China) were cultured, and exosome isolation was performed as described previously with minor modifications in Fig. 1 [9, 18]. All animal procedures were approved by the Ethics Committee of Tianjin Medical University and were in accordance with the Guidance Suggestions for the Care and Use of Laboratory Animals. First, the SCs were cultured in exosome-depleted growth medium, and the cell conditioned medium was collected. Then, dead cells and large growth debris were removed through centrifugation (1000 × *g*, 20 min). Second, to remove additional debris, the supernatant was centrifuged at 10,000 × *g* for 30 min. Then, the supernatant was filtered using 0.22-µm filters (Millipore, CA). Then, the filtered supernatant was centrifuged at 100,000 × *g* for 70 min to collect the exosomes. Finally, the exosomes were resuspended in cold PBS for further experiments.

### Characterization of SCDEs by transmission electron microscopy (TEM) and the Malvern Zetasizer Nano ZS90

Transmission electron microscopy (TEM) was used for morphological observation. The exosome samples were prepared as described above. For TEM, briefly, the exosomes were fixed with 2.5% glutaraldehyde overnight at 4 °C. The solution was centrifuged at 100,000 × *g* to remove the glutaraldehyde, and the exosomes were washed three times with PBS. Then, the exosomes were stained with 3% phosphotungstic acid aqueous solution and fixed on copper mesh formvar grids. A transmission electron microscope (JEM-1010) was used to detect the exosomes. A Malvern Zetasizer Nano ZS90 (Malvern, UK) was used to detect the concentration of exosomes in different samples. Samples with appropriate concentrations were used to examine the size distribution of exosomes.

### Western blot

Exosome samples were lysed with RIPA buffer, and the collected protein samples were added to 5 × SDS loading buffer and denatured by boiling for 5 min. Then, 10% acrylamide gels were used for electrophoresis, and the proteins were transferred to PVDF membranes for 2 h. After washing three times with TBST, the membranes were blocked with blocking buffer (5% nonfat milk in PBS) for 1 h at room temperature (RT). Then, the membranes were incubated with primary antibodies overnight at 4 °C. The antibodies were diluted as follows: CD9 (rabbit monoclonal 1:2000; Abcam), Alix (mouse monoclonal 1:1000; Cell Signaling Technology) and TSG101 (rabbit monoclonal 1:500; Abcam). The next day, the membranes were washed three times with TBST and incubated with secondary antibody (1:2000) for 1 h at RT. Blots were detected using enhanced chemiluminescence.

### Protein digestion

Briefly, the obtained exosomes were lysed by 4% SDS, 100 mM DTT, and 50 mM Tris, and HCl (pH 7.5). According to the FASP protocol, trypsin was used to digest total proteins (20 µg). The proteins were denatured for 5 min at 95 °C and mixed with 200 mL UA buffer solution (8 M urea in 0.1 M Tris HCl pH 8.5) and centrifuged (12,000 rpm) three times for 15 min. Next, the samples and 100 mL of iodoacetamide (IAA) were mixed at 600 rpm in a hot mixer. The filter unit was incubated for 20 min and centrifuged for 10 min at 12,000 rpm. One hundred milliliters of UA was added to the filter unit and centrifuged at 12,000 rpm three times for 15 min. Then, 100 mL of 50 mM NH_4_HCO_3_ was added to the filter unit and centrifuged at 12,000 rpm three times for 15 min. Then, the samples were mixed with trypsin (with 50 mM NH_4_HCO_3_) and placed in the thermal mixer at 600 rpm. Then, these units were incubated in a wet chamber at 37 °C overnight. The filtration unit was transferred into the new collecting pipe and centrifuged for 15 min at 12,000 rpm. Then, 50 mM NH_4_HCO_3_ was added to the filter unit and centrifuged for 15 min at 12,000 rpm. Then, the samples were dried and stored at − 20 °C until LC-MS/MS.

### LC-MS/MS

Referring to a previous study [20], LC-MS/MS was performed with some modifications. In short, the final concentration of the samples was 0.1% (V/V) after dilution with formic acid. The samples were then loaded onto a 75 mm x 150 mm fused silica column that was packed in-house with 3-mm ReproSil-Pur C18 beads (120 Å; Dr. Maisch GmbH, Ammerbuch, Germany). LC-MS/MS was performed using an Easy Nano-UPLC 1000 (Thermo Electron, Waltham, MA). A gradient (5–80% acetonitrile and 0.1% formic acid) was used to elute peptides on the Q-Exactive mass spectrometer (Thermo Electron, Waltham, MA) in the flow rate of 300 l/min over 240 min. The MS/MS spectrum was obtained in the data-dependent mode, and the scan resolution was 70,000 during acquisition (m/z 200).

### Data processing

Raw data files were processed with Proteome Discoverer (v1.3; Thermo Scientific).

Then, Mascot 2.3.02 (Matrix Science) Boston, MA, software was used to search against the Bovine RefSeq database. Two missed cleavages are allowed for tryptic enzymes. Carbamidomethylation (C) was set to fixed modification, and oxidation (M) and acetylation (N-term) were set as variable modifications. The peptide difference was 20 ppm, the fragment ion mass error was 0.1 Da, and the peptide and protein identification error rates were < 1%.

### Gene Ontology annotation and pathway analysis

Functional-enrichment analysis for Gene Ontology (GO) terms was conducted through the Database for Annotation, Visualization and Integrated Discovery (DAVID, v6.8) (https://david.ncifcrf.gov/) with the entire murine genome as the background. The enriched GO analysis of annotated proteins was performed for cellular components, molecular functions and biological processes. Pathways enriched with the proteins were generated by KEGG pathway analysis and Cytoscape software 3.5.1. The Venn diagrams web tool (http://bioinformatics.psb.ugent.be/webtools/Venn/) was used to compare the proteins identified with the ExoCarta database. GraphPad Prism 6.0 was used for plotting.

## Results

### Characterizations of Schwann cell-derived exosomes

**Fig. 2:**
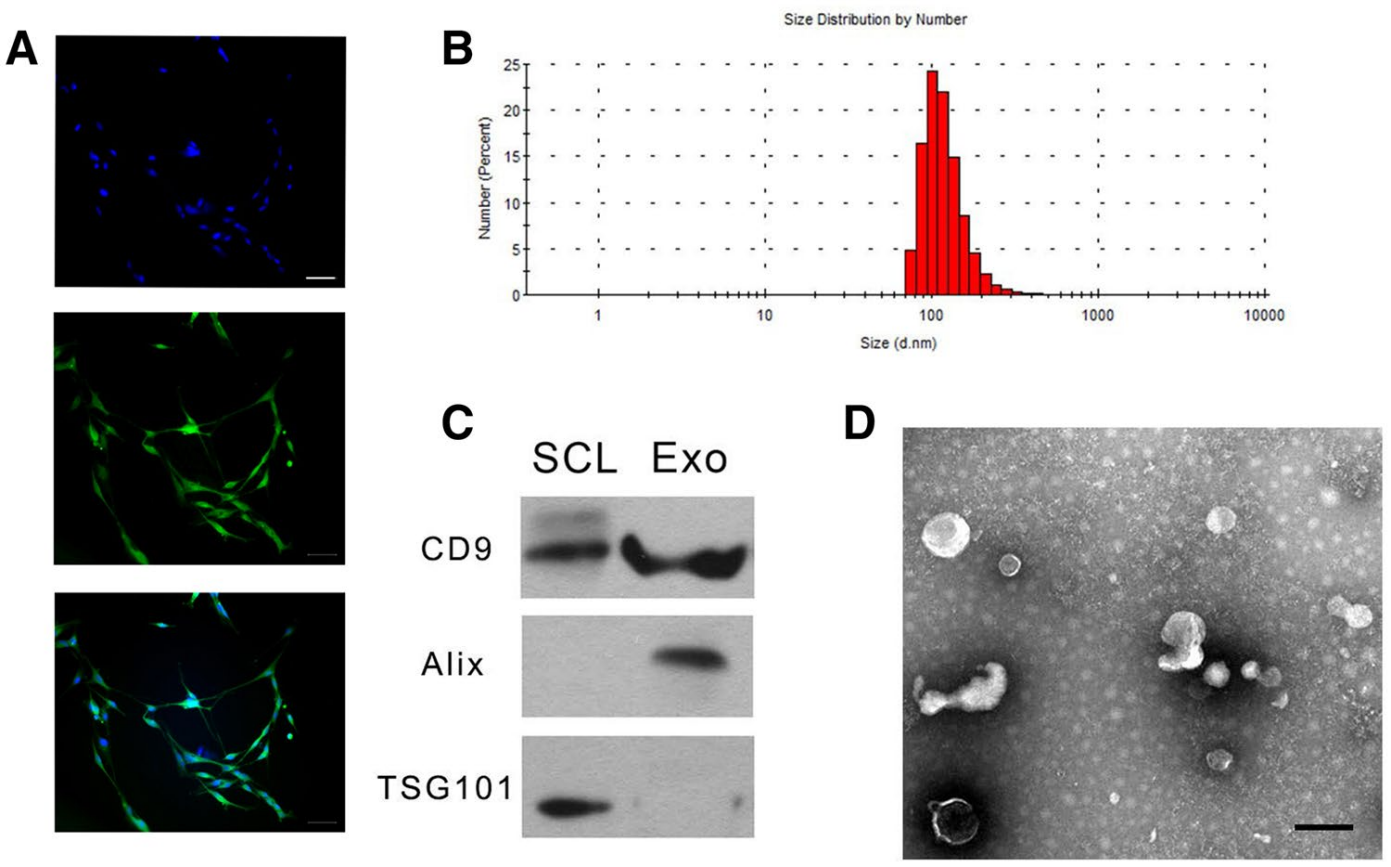
Characterizations of Schwann cell-derived exosomes. Characterization of the isolated SCDEs. **a** Schwann cells were positive for S100. (Bar = 50 µm). **b** The size distribution of SCDEs was detected by the Malvern Zetasizer Nano ZS90. **c** The expression of CD9, Alix, and TSG101 was detected by Western blot in the Schwann cell lysate (SCL) group and Schwann cell-derived exosome (SCDE) group. **d** TEM image of SCDEs

To explore the neuroprotective effect of SCDEs, we isolated exosomes from cultured Schwann cells. Schwann cells were positive for S100 (Fig. 2a). After observation using the Malvern Zetasizer Nano ZS90, the mean diameter of the SCDEs was 106.5 nm (Fig. 2b). We performed Western blotting to detect CD9, Alix and TSG101 expression to characterize these exosomes. The results showed that CD9 and Alix, which are exosome markers, could be detected, but TSG101 was not (Fig. 2c), which is consistent with exosome characteristics [18]. In addition, the morphology of SC-released exosomes was further confirmed through TEM, which showed SCDEs with a size range of 40–100 nm (Fig. 2d).

### Proteomic analysis of Schwann cell-derived exosomes

**Fig. 3:**
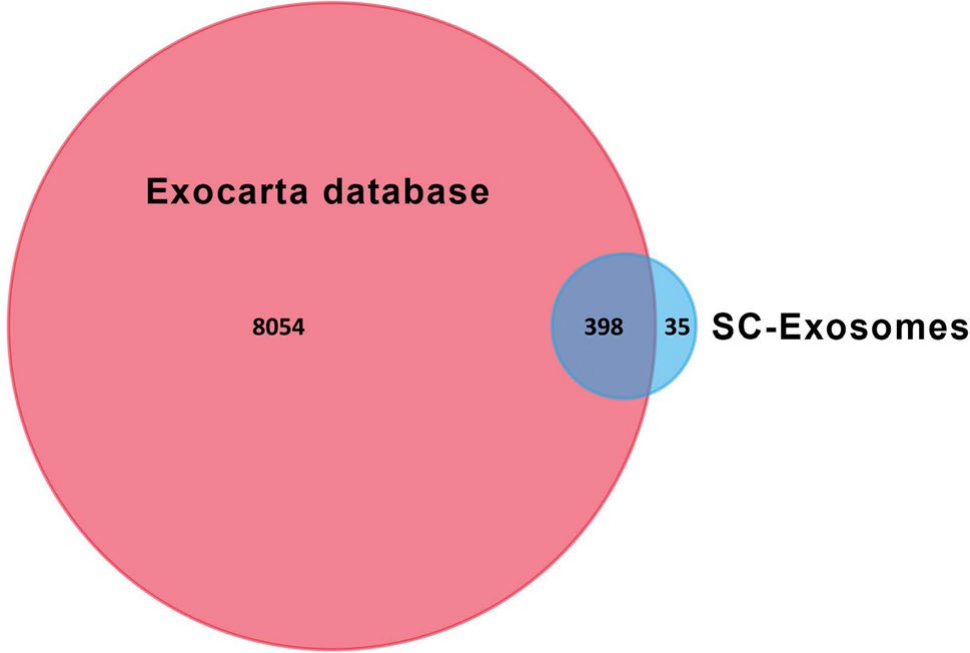
Venn diagrams of SCDEPs against the database of ExoCarta

We repeated the experiments three times (Supplementary Figure). To reveal the mechanisms of the neuroprotective effect of SCDEs, we performed proteomic analysis on SCDEs. Finally, 433 proteins in exosomes that were concordant in the three biological duplicates were identified and compared with the exosome database, ExoCarta; 398 proteins overlapped (Fig. 3) and were ranked based on intensity values (Supplementary Table). Thus, 91.92% of the identified proteins overlapped with ExoCarta, which indicated that the procedures for isolation and purification are repeatable, and the results of proteomic analysis are reliable. As shown in Table 1, most of the identified proteins are related to CNS repair.

**Table 1:** Exosome proteins associated with repair of SCI

Protein name	Protein IDs	Gene names	Role in repair of SCI
Carboxypeptidase E	P15087	CPE	Axon regeneration [21, 22]
Fatty acid-binding protein	P55053	FABP5	Axon regeneration [23, 24]
Fibronectin	P04937	FN1	Axon regeneration [25, 26]
Flotillin-2	Q9Z2S9	FLOT2	Axon regeneration [27–29]
Major vault protein	Q62667	MVP	Axon regeneration [30]
Monocarboxylate transporter 1	P53987	SLC16A1	Axon regeneration [31, 32]
Neuropilin-2	O35276	NRP2	Axon regeneration [33, 34]
Septin-7	Q9WVC0	SEPT7	Axon regeneration [35, 36]
Protein disulfide-isomerase A3	P11598	PDIA3	Axon regeneration [37–39]
Syntenin-1	Q9JI92	SDCBP	Axon regeneration [40]
αB-Crystallin	P23928	CRYAB	Inhibit inflammation [41]
Galectin-1	P11762	LGALS1	Inhibit inflammation [42, 43]

### SCDEs might enhance axon regeneration

Several studies have reported that SCDEs play a critical role in axonal regeneration in the PNS [18, 44, 45]. However, whether SCDEs enhance axon regeneration in the CNS is still unclear. According to the proteomics results, twelve proteins were closely related to axon regeneration, such as carboxypeptidase E (CPE), fatty acid-binding protein (FABP5), fibronectin, flotillin-2, major vault protein (MVP), monocarboxylate transporter 1 (MCT1), neuropilin-2 (NRP2), septin-7 (SEPT7), protein disulfide-isomerase A3 (PDIA3) and syntenin-1. As SCDEs contain proteins involved in axon regeneration, this result reveals that the function of SCs in promoting axonal regeneration might be through exosomes.

### SCDEs might inhibit inflammation

Exosomes from several kinds of cells participate in the inhibition of the inflammatory response, which is considered a novel therapeutic approach for some diseases [46–48]. Likewise, two proteins that we found in SCDEs, αB-crystallin and galectin-1, might produce benefits similar to anti-inflammatory effects in CNS injury. Thus, SCDEs might have an anti-inflammatory role in CNS damage.

### Functional categories

**Fig. 4:**
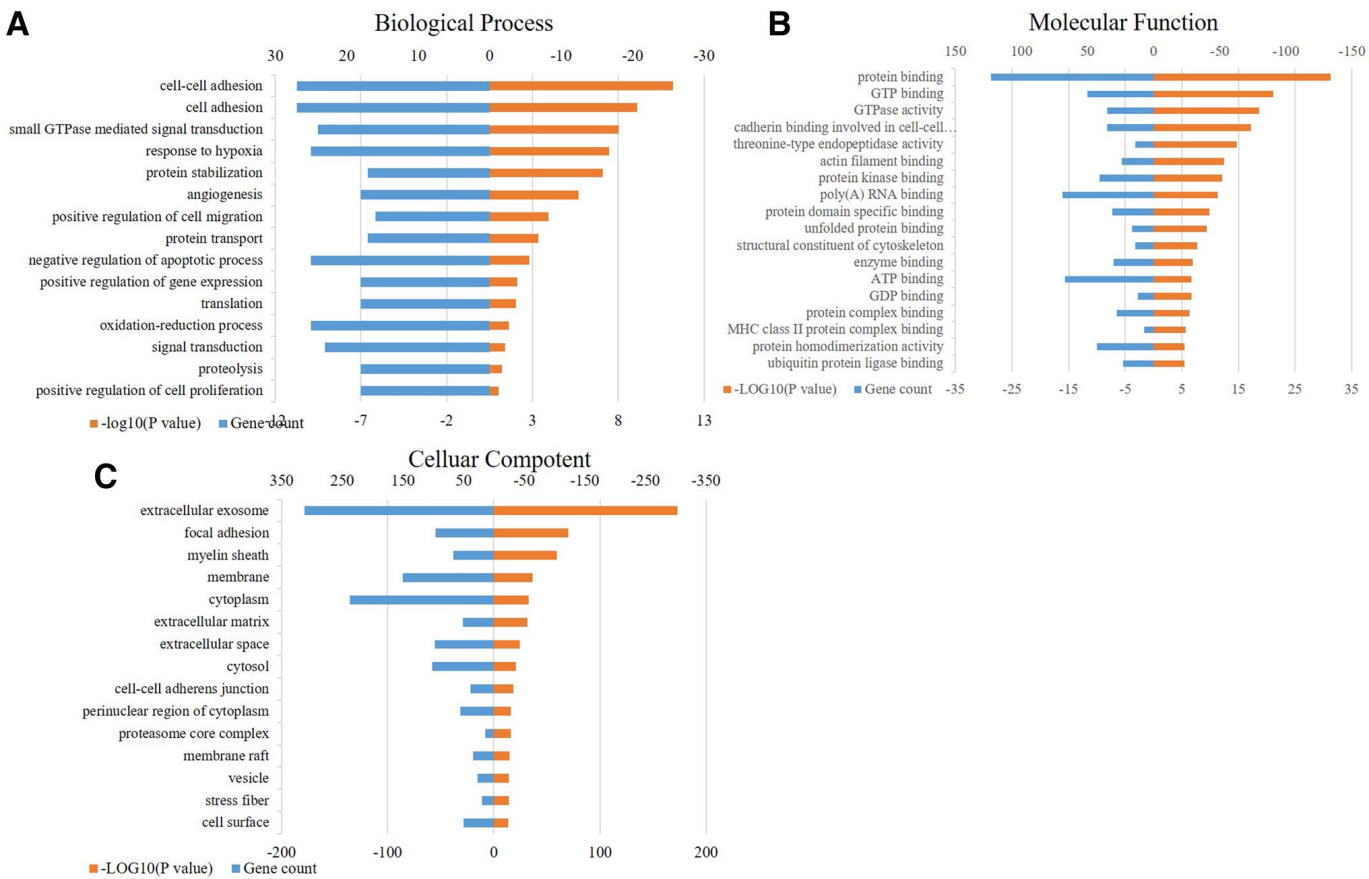
Gene Ontology (GO) analysis of biological processes, cellular components and molecular functions

Next, GO annotation was performed to determine the functional roles of the SCDE proteins (SCDEP) via DAVID version 6.8 and to obtain the enriched terms for molecular function, biological process and cellular component. The GO classification system revealed that the proteins could be classified into groups according to their functional properties (Fig. 4a–c). The biological processes analysis revealed enrichment of SCDEP related to “cell adhesion”, “negative regulation of apoptotic” and “signal transduction”. For the cellular component, these proteins were enriched in “exosomes”, the “cytoplasm” and the “membrane”. The molecular functions of the exosome proteins are mainly enriched in “protein binding”, “poly (A) RNA binding” and “GTP binding”.

### KEGG pathway analysis

The enriched signaling pathways with the exosome proteins were determined using KEGG pathway analysis. The top 20 pathways based on enrichment were given. As shown in Table 2, the top 20 pathways enriched with a p value < 0.05 were focal adhesion, endocytosis, regulation of the actin cytoskeleton and the PI3K-Akt signaling pathway. Among these, the neurotrophin signaling pathway, PI3K-Akt signaling pathway and cAMP signaling pathway are related to the CNS microenvironment.

**Table 2:** KEGG pathway analysis

Number	Term	Count	*P* Value
1	rno04510: Focal adhesion	26	2.13E-07
2	rno04144: Endocytosis	26	5.92E-05
3	rno04810: Regulation of actin cytoskeleton	24	5.46E-06
4	rno04151: PI3K-Akt signaling pathway	21	0.0269219
5	rno03010: Ribosome	20	1.91E-05
6	rno04145: Phagosome	19	3.44E-04
7	rno04015: Rap1 signaling pathway	18	0.002744107
8	rno04024: cAMP signaling pathway	15	0.013664262
9	rno04921: Oxytocin signaling pathway	12	0.035715206
10	rno04141: Protein processing in endoplasmic reticulum	12	0.048063015
11	rno04062: Chemokine signaling pathway	12	0.065106331
12	rno00480: Glutathione metabolism	11	4.59E-05
13	rno04915: Estrogen signaling pathway	11	0.002866544
14	rno04270: Vascular smooth muscle contraction	11	0.015103761
15	rno04360: Axon guidance	10	0.048391286
16	rno04918: Thyroid hormone synthesis	9	0.003507414
17	rno04520: Adherens junction	9	0.00543844
18	rno04512: ECM-receptor interaction	9	0.017206177
19	rno04722: Neurotrophin signaling pathway	9	0.096478076
20	rno04961: Endocrine and other factor-regulated calcium reabsorption	7	0.007433825

## Discussion

Schwann cells are the main functional glial cells in the PNS and play a very important role in axon regeneration after PNS injury. Recent studies believe that Schwann cells and their encapsulated axons should be deemed a “functional syncytium” [44] because of the inadequate capability of overlong axons to transport enough proteins in time and the evidence of substance exchange between axons and Schwann cells. Exosomes are small vesicles carrying functional molecules from host cells and play an important role in cell–cell communication [49]. Exosomes derived from Schwann cells modulate the damaged PNS microenvironment and enhance axonal regeneration by inhibiting the activity of the GTPase RhoA [18]. This finding gives us a new promising strategy for other neurodegenerative disorders, such as amyotrophic lateral sclerosis (ALS), Alzheimer’s disease (AD) and brain and spinal cord injury. Therefore, there is an urgent need to investigate exosome contents, which can help us gain a deeper understanding of potential therapeutic mechanisms. In this study, we isolated SCDEs and verified the size, morphology and surface markers associated with exosome characteristics. Then, proteomics analysis was performed, and 433 proteins were identified through LC-MS/MS. Ultimately, bioinformatics analysis was performed to reveal their potential mechanism.

The Schwann cells in this study were isolated from the sciatic nerves of rats according to our previous methods, and by doing so, the purity of Schwann cells was more than 95% [50]. Until now, there has been no advanced method to purify cultured Schwann cells to 100%, and fibroblast contamination is difficult to avoid [51]. Despite the existence of the infinite cell line RSC96, which acts as an alternative for primary Schwann cells in some aspects [52], comparative proteomic analysis confirmed that this cell line exhibited different secreted protein expression profiles [53]. In this study, we purified Schwann cells to approximately 100% purity, which can give us reliable results.

The GO analysis of proteomics data revealed that the proteins in SCDEs were enriched in cellular processes, cellular processes involving biological regulation, which indicated that the contents of exosomes share close ties with microenvironment regulation. This regulatory characteristic is consistent with the supporting cell status for Schwann cells [54]. In the cellular component of GO analysis, the enrichment of cytoplasm and cytosol reflects the origin of exosomes. In addition, membrane-bound vesicles and plasma membranes are consistent with exosome features. For molecular function, protein binding is the most common characteristic, revealing that direct regulation of protein–protein interaction might be the main kind of regulation for SCDEs.

We used the KEGG pathway database to explore the enrichment of proteins and found that several signaling pathways were related to proteins in SCDEs. Some pathways reflect the basic biofunction of cells, such as the proteasome, glycolysis and regulation of the actin cytoskeleton, which proved that exosomes act as a suborgan of the host cells. We noticed that some proteins were enriched in the ribosome pathway, which is consistent with the fact that Schwann cells can transfer ribosomes to nearby axons and confirms that Schwann cells play an important role in supporting local protein synthesis in axons [55]. In the future, we will explore whether regeneration-related mRNAs exist in such exosomes.

Considering the potential therapeutic ability of CNS, we found that several enriched pathways are related to the CNS microenvironment, including the neurotrophin signaling pathway, PI3K-Akt signaling pathway and cAMP signaling pathway. In the neurotrophin signaling pathway, we found that Rho GTPases were detected, especially Rac1 and Cdc42. The Rho family of GTPases belongs to the Ras superfamily and plays an important role in neuronal development, neuronal survival and neurodegeneration [56]. In the CNS, Rac1 and Cdc42 can promote neurite outgrowth and stimulate regeneration [57]. In contrast to neurons, astrocytes are always activated and form glial scars to inhibit axon regeneration [58]. The high expression of Rac1 can inhibit astrocyte outgrowth [59]. Meanwhile, Rac1 and Cdc42 can cause astrocyte apoptosis induced by neurotoxicity [60]. In addition, activation of Rac1 and Cdc42 can change microglia into the M2-like phenotype, which is beneficial in CNS regeneration [61]. Analogously, Rac1/Cdc42 signaling can promote the migration of oligodendrocytes, the key cells in CNS for myelinization [62]. A recent study also proved that the activation of Rac1 can preserve blood-spinal cord barrier (BSCB) integrity and improve functional recovery after spinal cord injury through protection of endothelial cells [63].

## Conclusions

The proteins in SCDEs can create a more permissive microenvironment related to each component in the CNS for regeneration. Our proteomics analysis may provide a novel therapeutic strategy for CNS injury.

## Electronic supplementary material

Below is the link to the electronic supplementary material.

Supplementary material 1 (PNG 68 KB)

Supplementary material 2 (XLSX 28 KB)
