# Supplementary figures and images for: Proteomics analysis of Schwann cell-derived exosomes: a novel therapeutic strategy for central nervous system injury
Source: Mol Cell Biochem. 2019 Mar 4;457(1):51–9. doi: 10.1007/s11010-019-03511-0 (PMC6548868; doi:10.1007/s11010-019-03511-0)

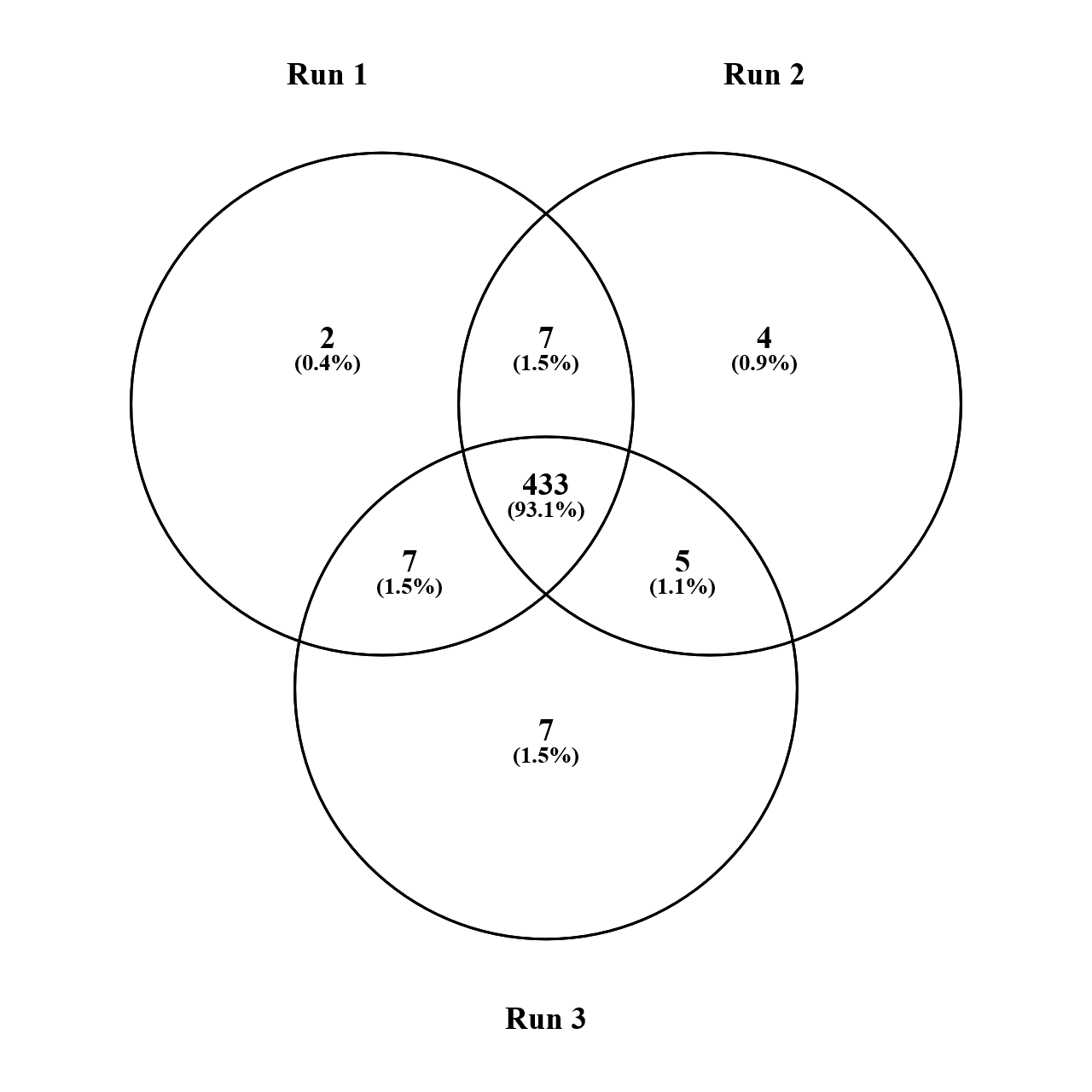

Supplement: Supplementary file 1 — Supplementary material 1 (PNG 68 KB) [file 11010_2019_3511_MOESM1_ESM.png]
